# An Isogenic Yeast Model Reveals the Influence of Native Promoter G‑Quadruplex Forming Sequences on P53 and NF-κB Transactivation

**DOI:** 10.1021/acsbiomedchemau.6c00109

**Published:** 2026-07-04

**Authors:** Libuše Kratochvilová, Petr Orság, Karolína Drápalová, Radim Stříž, Zeinab El Rashed, Paola Menichini, Rieko Ohki, Paola Monti, Alberto Inga, Václav Brázda

**Affiliations:** † Institute of Biophysics of the Czech Academy of Sciences, Královopolská 135, 61200 Brno, Czech Republic; ‡ Department of Food Chemistry and Biotechnology, Faculty of Chemistry, 649431 Brno University of Technology, Purkyňova 118, 61200 Brno, Czech Republic; § Gene Expression Regulation, IRCCS Azienda Ospedaliera Metropolitana, 16132 Genoa, Italy; ∥ Neuro-oncology and Mutagenesis, IRCCS Azienda Ospedaliera Metropolitana, 16132 Genoa, Italy; ⊥ Laboratory of Fundamental Oncology, National Cancer Center Research Institute, 5-1-1 Tsukiji, Chuo-ku, Tokyo 104-0045, Japan; # Laboratory of Transcriptional Networks, Department of Cellular Computational and Integrative Biology, CIBIO, University of Trento, via Sommarive 9, 38123 Trento, Italy

**Keywords:** G-quadruplex forming sequences (G4FS), G4-quadruplexes (G4s), transcription regulation, transcription factors (TFs), p53 family proteins, NF-κB family proteins, yeast isogenic system

## Abstract

G-quadruplexes (G4s) are noncanonical, four-stranded nucleic acid structures formed in guanine-rich regions that influence many biological processes, including transcription. We previously developed a yeast-based system to study the transcriptional modulation of p53 family proteins by G4-forming sequences (G4FS) using artificial constructs. Here, motivated by the observation of genome-wide enrichment of G4FS in proximity to p53 and NF-κB response elements (REs), we examined how native combinations of G4FS and REs derived from human promoters influence the activity of p53 or NF-κB family proteins. To this end, five pairs of isogenic reporter strains were developed, and the propensity of G4FS to adopt G4 structures was confirmed by *in vitro* assays. Results in yeast showed that the presence of G4FS enhanced the transcriptional output of partial-function p53 mutants, p63, and p73 relative to wild-type p53, suggesting a general boost in the activity of weaker transcription factor proteins. Conversely, the effect of G4FS on transactivation by NF-κB proteins was context-dependent and mainly inhibitory. In conclusion, our findings highlight the importance of DNA topology in transcriptional regulation by both p53 and NF-κB proteins and demonstrate that the yeast-based system serves as a valuable tool for isolating the contribution of G4FS within a controlled genomic context.

## Graphical abstract

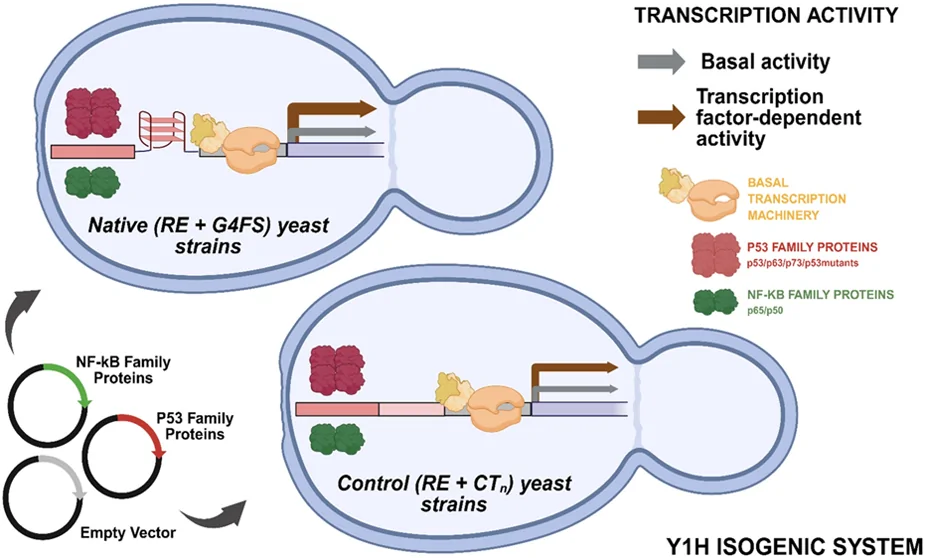

## Introduction

Protein–DNA interactions play a fundamental role in regulating gene expression, influencing cellular responses, and maintaining genomic integrity. Increasing evidence suggests that local DNA secondary structures, particularly G-quadruplexes (G4s) are emerging as key regulatory elements.^1^ The occurrence of G-quadruplex forming sequences (G4FS) has been observed in genomic regions with significant regulatory functions, including gene promoters, telomeric regions, introns, and untranslated regions (UTRs) of mRNA, as well as replication initiation sites.^2^

The presence of G4s has a significant impact on a number of fundamental biological processes, including DNA replication, transcription, and mRNA translation; therefore, strict regulation of their formation is essential within the cell.^3^ G4s present in gene promoters and enhancers, or localized just downstream of the transcription start site (TSS), can support transcription; transcription activation is mediated by the recognition of G4s as target motifs, which serve as platforms for the recruitment of protein complexes that activate transcription.^4^ Recent studies have further demonstrated that both DNA and RNA G4s behave as stress-responsive regulatory elements, whose folding and stability are dynamically modulated under cellular stress conditions, thereby shaping transcriptional and post-transcriptional gene regulation. Notably, G4 motifs are enriched in stress-response genes and can act as regulatory hubs that integrate DNA topology, chromatin remodeling, and transcription factor activity during adaptive stress responses.^5,6^

The p53 and Nuclear Factor-kappa B (NF-κB) families of TFs are among the most studied proteins in tumor biology, typically known to function as antagonists, although recent studies have identified examples of positive, even cooperative interactions.^7^ Both p53 and NF-κB proteins act as dimers or as dimers of dimers, and feature relatively large DNA-binding domains comprising a β-sandwich fold and acidic transactivation domains, and bind specific response elements (REs), multiple versions of which exist in the genome, coordinating extensive networks of target genes.^8,9^

The p53 family includes p53, as well as p63 and p73.^10^ The p53 protein is referred to as the “guardian of the genome”, and in response to stress, DNA damage, or aberrant proliferation, it mediates processes such as cell cycle arrest, DNA repair, apoptosis, cellular senescence, ferroptosis, autophagy, and the maintenance of metabolic homeostasis.^11,12^ While p53 functions as a sensor of DNA damage and oxidative stress, p63 and p73 proteins are essential for maintaining cellular homeostasis.^13^ Both p63 and p73 as full-length isoforms, due to their shared structural features, can regulate p53 target genes by binding to the same consensus sequence (i.e., RRRCWWGYYY-(n)-RRRCWWGYYY, where R = purine; W = A/T; Y = pyrimidine; separated by *n* = 0–13 base pairs)^14^ and partially compensate for the loss of p53, which is observed in over 50% of malignancies.^15^ Intermolecular interactions among p53 family proteins are currently being studied due to their overlapping roles in the suppression of cancer development and in the maintenance of epidermal homeostasis.^16^

Interestingly, it has been shown that the p53 protein can recognize and bind G4FS in selected promoters (e.g., *c-MYC*, *VEGF*), even with higher affinity than to its classical consensus binding sequences.^17,18^ The p53 protein can use G4s as alternative recognition motifs to regulate transcription, allowing the control of genes that lack classic consensus binding motifs.^18^ p53 mutations can also be affected by the presence of G4 structures; indeed, a high frequency of wild-type and R282W p53 ChIP-seq bound peaks that overlap with predicted G4 sequences was observed in the K562 myeloid leukemia cell line.^19^ In addition to p53 and its mutants, p63- and p73-dependent transactivation can also be modulated by G4FS.^20^

The NF-κB family of TFs plays a central and evolutionarily conserved role in regulating gene expression programs essential for innate and adaptive immunity, inflammation, and cell survival.^21^ This family comprises the five structurally related members—RelA (p65), RelB, c-Rel, NF-κB1 (p105/p50), and NF-κB2 (p100/p52),^22,23^ which form various homo- and heterodimeric complexes. NF-κB binds to specific consensus motifs (i.e., GGGRNNYYCC, where R = purine; N is any nucleotide; Y = pyrimidine)^24^ within the promoters and enhancers of a broad array of target genes that mediate immune responses, cell proliferation, and antiapoptotic signaling.^25^ The potential involvement of G4 structures in NF-κB-dependent transcriptional regulation remains largely unexplored. Nevertheless, bioinformatic analyses indicate that several genes involved in the inflammatory response, including NF-κB signaling, exhibit significant enrichment of sequences predicted to adopt G4 structures in regulatory regions.^26^

In this study, we investigated how native G4FS from human promoters of p53 and NF-κB target genes modulate the transcriptional activity of both protein families using a well-established functional assay in the yeast *Saccharomyces cerevisiae*. These functional studies were motivated by a genome-wide association of G4 motifs with p53 and NF-κB binding sites as well as by the confirmation of their propensity to adopt G4 structures *in vitro.* Specifically, we focused on three p53-regulated genes, *BBC3* (PUMA), *ASTN2*, and *TRIM32*, which represent distinct functional classes of p53 target genes relevant to cancer biology. For NF-κB, the two target genes IκBα and RelB, involved in feedback inhibition and dimer stoichiometry, respectively were chosen.^27,28^ These genes contain a weak (IκBα) and a strong (RelB) binding site, but G4FS on the opposite strand relative to the direction of transcription. By analyzing these promoters, we aimed to capture how promoter context and DNA topology influence the transcriptional output of p53 family members, clinically relevant p53 mutant proteins with distinct residual activities, and NF-κB family proteins.

## Materials and Methods

### Prediction of Human p53 and NF-κB Binding Sites with G4FS

The genomic data utilized in this study were obtained from the National Center for Biotechnology Information (NCBI) genome database. We employed the human reference genome assembly Genome Reference Consortium Human Build 38 (GRCh38), which offers a comprehensive and high-quality representation of the human genome, including essential annotations; all chromosome sequences were downloaded in FASTA format and subsequently used for downstream analyzes. G4FS were identified using the G4Hunter tool,^29^ which evaluates the guanine content and distribution within DNA sequences to predict regions with a high propensity to form G4 structures. For this analysis, a G4Hunter score threshold of ≥ 3.0 was applied to ensure the inclusion of only those sequences with strong G4-forming potential and high biological relevance; this stringent cutoff minimizes false positives and focuses the analysis on stable and functionally significant G4 motifs. All predictions were performed under default parameter settings, and the resulting G4FS data were compiled for each chromosome. To assess the potential functional relevance of predicted G4FS, we conducted a genome-wide colocalization analysis using the ChIP-seq data sets. Genomic coordinates of Layer 1 G4FS predictions were formatted in BED format and mapped to the GRCh38 human genome assembly. The analysis was performed using the ChIP-Atlas Enrichment Analysis tool (https://chip-atlas.org),^30,31^ which enables statistical evaluation of genomic feature overlap across thousands of ChIP-seq experiments. We tested all p53 and NFκB1 (Nuclear Factor Kappa B Subunit 1, precursor of the active p50 subunit) entries in the ChIP-Atlas database. We applied a stringent significance threshold of 100 for ChIP-seq peak intensity and incorporated 100 permutations of the input data set to construct a robust background model. Summary statistics and detailed outputs are provided in the Supporting Material 01.

### Characterization of p53 and NF-κB-Associated G4FS by ThT Assay and CD Spectroscopy

The native G4FS from *BBC3 (PUMA)* promoter was used together with short flanking sequences to preserve the native promoter context, and was analyzed alongside the KSHV G4 sequence as a control (Table 1); both sequences have been previously studied as natural and artificial p53 RE-associated G4s, respectively.^20^ Importantly, the inclusion of flanking nucleotides in all oligonucleotides under analysis was intentionally chosen to approximate the natural genomic environment of the promoter-embedded G4 motifs, as these neighboring sequences are known to influence G4 folding propensity, topology, and stability. Oligonucleotides with G4FS derived from the gene promoters upregulated by p53 (i.e., *ASTN2* and *TRIM32*)^19^ or by NF-κB (i.e., *RelB* and *IκBα*) (Table 1) were dissolved in deionized water at a concentration of 100 μM and further processed. This strategy overcomes our recent studies employing minimal, systematically engineered constructs^20^ and was adopted here to enable the functional interrogation of G4FS in a sequence context that closely resembles the natural promoter setting. To perform the ThT (Thioflavin T) assay, the oligonucleotides were denatured at 95 °C for 5 min and slowly cooled to room temperature. Samples were then analyzed in Tris-HCl buffer alone (cation-deficient control) or in the same buffer supplemented with 100 mM KCl, 10 mM NaCl, or 100 mM LiCl, the latter serving as a standard negative control for canonical G-quadruplex stabilization. An equimolar volume of 1 μM ThT was added to the oligonucleotide samples (resulting DNA:ThT concentration ratios were 0.1:0.5). ThT is a fluorescent probe developed to identify amyloid fibrils,^32^ whose ability to recognize G4 sequences was subsequently confirmed.^33^ Fluorescence intensity was measured in a 384-well microtiter plate on an Agilent BioTek Synergy H1 microplate reader. The fluorescence intensity of the sample was normalized to that of the ThT buffer alone, which also served as a negative control; 100 mM KCl or 10 mM NaCl was added to stabilize the G4 structure.^34,35^ Samples were measured in triplicate, and the averages of the determined I/I_0_ and standard deviation values were calculated.

**Table 1:** G4FS Analyzed in the Present Study

G4FS	Sequences 5′–3′	Length [bases]	G4Hunter score [-]
KSHV G4	GGGGCGGGGGACGGGGGAGGGG	22	3.182
TRIM32 G4	TGGGGAGGCGGGGCTCAGTGACGGACAGGGA	31	1.484
BBC3 G4	ACTTGTCCGCGGCGGGCGGGCGGGG	25	1.240
ASTN2 G4	CGGGGCAAGGCGGCCCTGCAGGGGTGA	27	1.160
RelB G4	GGGCGGGCGGCGGGCGGGGGCGGGG	25	2.207
IκBα G4	TGGGGCGGGGTCAGGCTCGGGGAATTTCC	29	1.517

Nondenaturing polyacrylamide gel electrophoresis (PAGE) was also used to analyze the formation of G-quadruplex structures. Six oligonucleotides containing G4-forming sequences derived from the native promoter contexts, the previously characterized KSHV-derived G4FS,^19^ and a random single-stranded DNA control sequence (22 nt) were analyzed at a final concentration of 10 μM. Samples were prepared in 10 mM Tris buffer in the absence or presence of 100 mM KCl, and loaded onto native 8% polyacrylamide gels either without added salt or supplemented with 10 mM KCl. Electrophoresis was performed at 4 °C to preserve secondary structures. DNA migration was monitored using the O’RangeRuler 10 bp DNA Ladder (ready-to-use); following electrophoresis, gels were sequentially visualized by staining with Thioflavin T (ThT, 0.5 μM), thiazole orange (2.73 μM) or Stains-All. Staining was carried out for 24 h, followed by destaining in water.

For CD (circular dichroism) analysis, DNA samples (60 μM) were prepared in 10 mM Tris-HCl or 10 mM Tris-HCl with the addition of 100 mM KCl or 10 mM NaCl to stabilize the G4 structure.^34,35^ Oligonucleotides were denatured at 95 °C for 5 min and slowly cooled to room temperature. Spectra were measured on a Jasco 815 circular dichroism spectrometer in 1 cm tapered quartz cuvettes that were placed in a thermostatically controlled holder at 23 °C. Four scans of each sample were taken at a scan speed of 100 nm/min with data spacing of 0.5 nm in the wavelength range 210–330 nm, averaged, and the resulting spectra smoothed using a Savitzky-Golay smoothing algorithm with a 25-point window. The CD signal was expressed as the difference in the molar absorption coefficient Δε of left and right polarized light.

### Yeast Reporter Assay

New isogenic yeast strains were prepared by the Delitto Perfetto site-specific mutagenesis method.^36^ *S. cerevisiae* ISce-I endonuclease under *GAL1* promoter (yLFM-ICORE), counter selectable *URA3*, and reporter *KanMX4* conferring kanamycin resistance yeast strains were modified to XV yeast chromosome upstream of the minimal yeast *pCYC1* promoter and the *LUC1* reporter gene derived from the firefly gene for luciferase expression.^37^ The iCORE sequence was replaced by sequences containing the RE from the p53 or NF-κB target gene plus a sequence with G4 potential formation (yLFM native strains, n = native); corresponding control strain were constructed by replacement of the G4 sequence with a cytosine and thymine repeat (CT_n_) (yLFM control strains, c = control). Then, the available strains (i.e., yLFM-nBBC3, yLFM-cBBC3, yLFM-nTRIM32, yLFM-cTRIM32, yLFM-nASTN2, yLFM-cASTN2, yLFM-nRelB, yLFM-cRelB, yLFM-nIκBα and yLFM-cIκBα) were transformed with tryptophan-selective pTSG expression plasmids (*GAL1,10* promoter) or empty vector (pRS314) by the lithium-acetate method. Vectors containing the coding sequences for wild-type p53 family TA α isoforms (i.e., p53, p63, and p73), p53 mutant proteins (i.e., A161T, N235S and R283C) or NF-κB proteins (i.e., p50 TAD and p65) were used. Bidirectional vectors coexpressing p50 and p65, or p50 TAD, a chimeric construct that contains the RelA transactivation domain that is active in yeast, and p65, were also available (Laboratory of Transcriptional Networks, CIBIO, University of Trento). Transformants were resuspended in selective medium containing galactose (1%), which induces protein expression, and were incubated at 30 °C with constant shaking; after 6 h, the established cultures were measured for OD_600_ and signal of bioluminescence (i.e., firefly units). OD_600_ measurements were used both to normalize luciferase activity and to assess potential effects of transcription factor overexpression on yeast growth. Comparison of OD_600_ values between strains expressing p53 family proteins (wild-type and mutants, p63, and p73) or NF-κB family proteins and the corresponding empty-vector controls revealed no significant differences in overall growth, indicating that protein overexpression did not measurably affect yeast viability or proliferation under the experimental conditions used. For the reporter assay, yeast cell suspension (10 μL) was taken from each sample and lysed with an equimolar volume of 2× passive lysis buffer (PLB 5×, Promega) with constant shaking at room temperature for 15 min. Luciferase substrate (10 μL, Bright Glo Luciferase Assay, Promega) was added to the lysed cells, and the bioluminescence value was immediately measured on the microplate reader Agilent BioTek Synergy H1. Luciferase activity was quantified using a multilevel normalization strategy to account for differences in cell density and basal promoter activity; raw luminescence values were first normalized to cell density (RLU = firefly units/OD_600_). Fold induction was then calculated by normalizing RLU values obtained upon specific transcription factor expression to the corresponding empty-vector control for each strain (i.e., RLU/RLU pRS314). This normalization allows comparison of transcriptional responsiveness independently of differences in basal reporter activity, which is particularly relevant for promoters containing native G4-forming sequences that display elevated baseline transcription. Relative activities were finally expressed as fold induction ratios, enabling direct comparison across strains and conditions. At least four independent cultures were analyzed for each experiment; data were analyzed by Student’s *t* test, followed by a comparison test, which was performed using GraphPad Prism version 8.0.1 (GraphPad Software, San Diego, California, U.S.A.).

## Results

### Analysis of G4FS Enrichment at p53 and NF-κB Binding Sites

First, we investigated the presence of G4FS near the binding sites of the p53 and NF-κB proteins in the human genome (see Materials and Methods section for details). We identified 27 NF-κB ChIP-seq and 206 p53 ChIP-seq experiments containing at least 1000 peaks (Supporting Information; Table S1); the enrichment of G4FS near ChIP peaks was statistically evaluated using a random list of sequences as a control. Both proteins’ ChIP-seq peaks are significantly enriched for the presence of potential G4FS; NF-κB data sets demonstrated a higher median enrichment (55.9-fold) than p53 (26.6-fold), indicating strong colocalization between NF-κB binding sites and potential G4FS (Figure 1).

**Figure 1:**
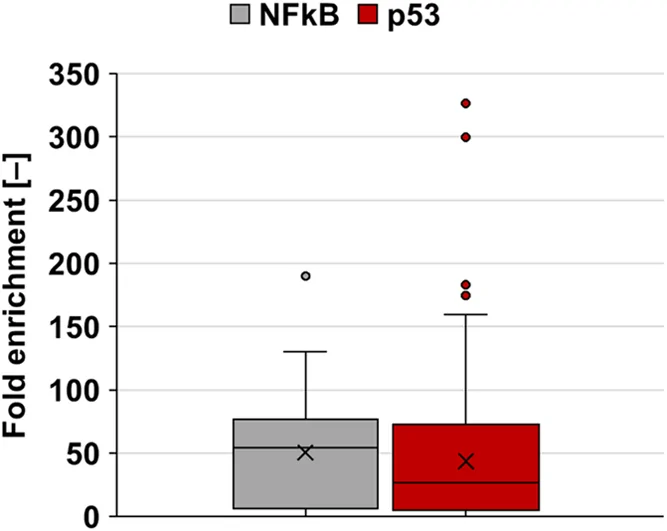
Enrichment of G4FS across NF-κb and p53 binding sites. Box plots represent the fold enrichment of G4FS within ChIP-seq peaks for NF-κB and p53 TFs. Each box summarizes the distribution of enrichment values across multiple ChIP-seq experiments retrieved from the ChIP-Atlas database.

### Selection of G4FS from p53 and NF-κB Target Genes

Guided by the enrichment for potential G4FS observed for p53 and NF-κB bound sites, we next decided to deepen the analysis on native promoter sites of a subset of target genes for both proteins. Regarding p53, we selected the G4FS derived from *BBC3 (PUMA)* promoter to further validate our previous studies,^20^ by including the functional analysis of all p53 family proteins (i.e., p53, p63, and p73) as well as three *TP53* mutations that differ in their residual transcriptional activity (i.e., A161T, N235S, and R283C); within p53 mutants we selected, N235S exhibited the highest transactivation activity, while R283C the lowest. The G4FS from the *TRIM32* promoter was selected since the G4FS is adjacent to the p53 RE similarly to the sequence derived from *BBC3* promoter while being characterized by a higher G4Hunter score (i.e., 1.484 for *TRIM32* G4FS vs 1.240 for *BBC3* G4FS) (Table 1); moreover, the *TRIM32* RE is a weaker p53-binding sequence with respect to the *BBC3* RE, the latter possessing a moderate strength. *TRIM32* has been identified as a gene coding for an E3 ubiquitin ligase that interacts with p53 and promotes p53 degradation through ubiquitination.^38^ Interestingly, this gene is nested within the *ASTN2* p53 target gene, coding for the neuronal protein astrotactin 2, and transcribed on the opposite strand with which it shares the p53 target RE; also, in the direction of *ASTN2* transcription a G4FS is found, downstream to the p53 RE and characterized by a lower G4Hunter score with respect to *TRIM32* G4FS (i.e., 1.160 for *ASTN2* vs 1.484 for *TRIM32*) (Table 1). Regarding NF-κB, we selected the G4FS from *IκBα* and *RelB* gene promoters, exploiting the possibility of modeling their functional impact in an isogenic, yeast-based transcriptional reporter system we already developed. Interestingly, the G4FS sequences for both NF-κB target genes are located on the opposite (negative) strand relative to the REs and the transcription start sites; the two sequences differ in distance with respect to RE sequence (i.e., adjacent in *IκBα* or downstream in *RelB*) as well as for their G4 propensity as determined by the G4Hunter score (i.e., 1.517 for *IκBα* G4FS and 2.207 for *RelB* G4FS). This selection led to *in vitro* analysis of three p53 and two NF-κB native sequences from corresponding target genes, where G4FSs are all located adjacent to or downstream of the TF RE.

### Biophysical Characterization of Selected G4FSs from p53 and NF-κB Target Genes

Based on the selection of the candidate G4FSs from p53 and NF-κB target genes, we first investigated the *in vitro* potential for G4 formation under physiological conditions. First, G4 formation was investigated by ThT assay (Figure 2A). During the ThT assay, the increase in fluorescence I/I_0_ of G4FSs was compared with respect to the positive control (i.e., KSHV G4), in which G4 formation has been demonstrated both in an environment with K^+^ ions and in one without stabilizing ions,^20^ relative to the negative control (i.e., buffer without DNA). The results from all tested oligos showed higher measured *I*/*I*_0_ values than the buffer without DNA itself, and the highest fluorescence values were measured for all native G4FS in the presence of K^+^ ions; the highest *I*/*I*_0_ value was achieved by TRIM32 G4, which showed an even higher increase than the control KSHV G4. In the presence of Na^+^ ions, there was a decreased, but still noticeable, formation of G4s, especially in RelB, IκBα, and TRIM32 G4s, although the concentration of Na^+^ ions used was 10-fold lower than the potassium ions. The formation of G4s was shown even in buffer without stabilizing conditions, especially for RelB G4 and TRIM32 G4 sequences. Importantly, the present results are based on relative fluorescence differences obtained from within-experiment comparisons using side-by-side measurements with the KSHV G4 control under identical conditions, rather than on absolute ThT fluorescence values across different studies, which are known to vary due to methodological and instrumental factors. To further validate the assignment of G4-specific signals and exclude potential ligand-induced or noncanonical structures, additional ThT assays were performed in LiCl buffer, which does not support canonical G-quadruplex stabilization (Supporting Information 02), and G4 formation was independently assessed by PAGE (Supporting Information 03).

**Figure 2:**
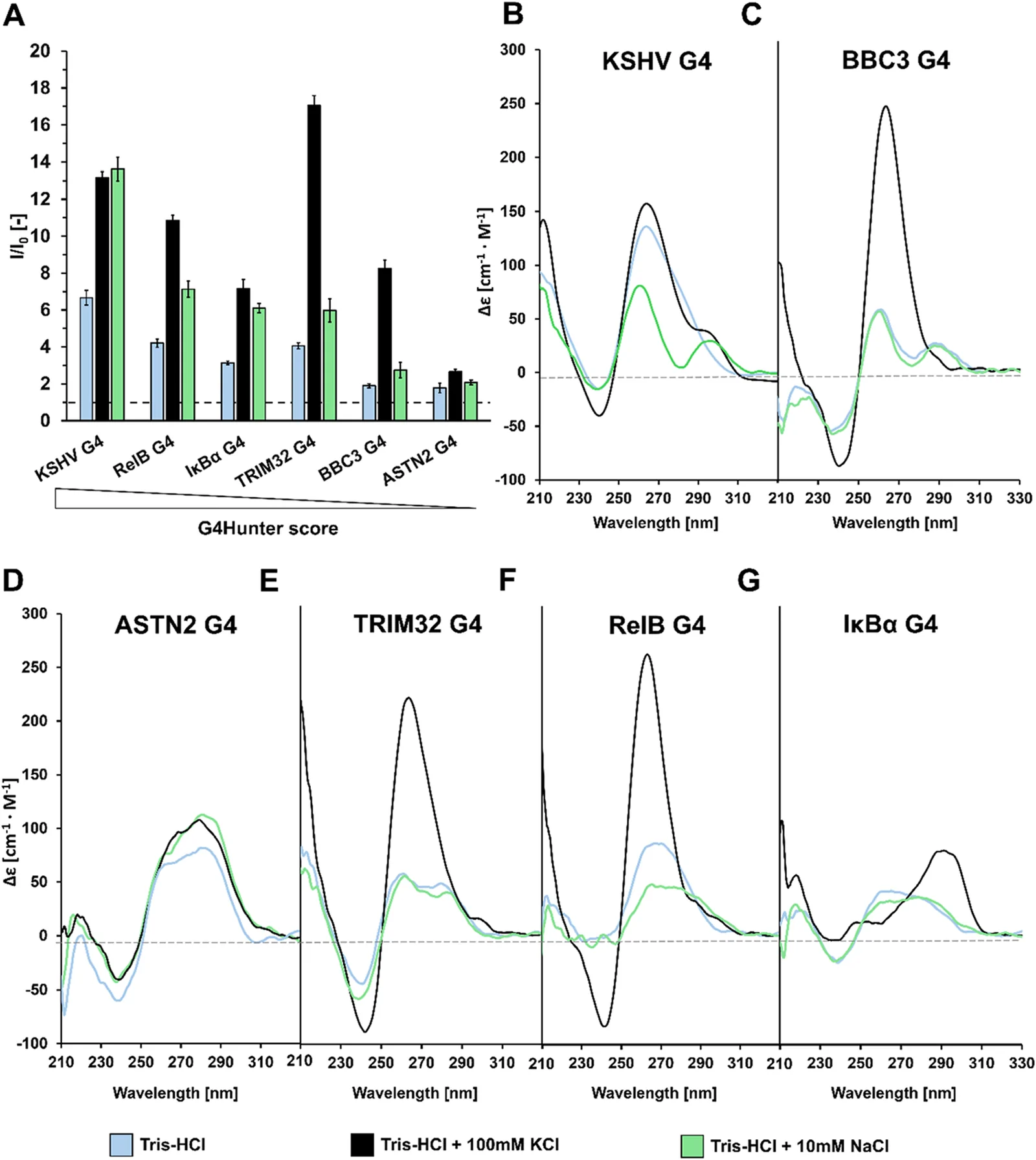
Biophysical characterization of G4 oligonucleotides. Oligonucleotides were hybridized in Tris-HCl (blue columns/lines) or in the same buffer supplemented with 100 mM KCl (black columns/lines) or 10 mM NaCl (green columns/lines). (A) ThT assay: the fluorescence intensity (I) was normalized to the fluorescence intensity of ThT buffer alone (*I*_0_). The black dashed line indicates the value of the buffer without DNA as a negative control. (B, C, D, E, F, G) CD spectroscopy: CD spectra of the indicated G4 oligonucleotides were measured in the wavelength range 210–330 nm, smoothed with a 25-point Savitzky-Golay algorithm, and the baseline of the DNA-free buffer was subtracted.

The conformation of the formed secondary structures was characterized by CD spectroscopy (Figure 2B–G); the oligonucleotides were dissolved in either the 10 mM Tris-HCl buffer (pH 7.5) or in Tris-HCl buffer with the addition of stabilizing 10 mM Na^+^ or 100 mM K^+^ ions, reflecting the usual ion concentrations in physiological intracellular conditions.^40,41^ The results clearly demonstrated the formation of parallel G4s conformations in KSHV G4, as expected, along with BBC3, RelB, and TRIM32 G4s in the environment of stabilizing K^+^ ions with characteristic positive peaks at 210, 260 nm and a negative peak at a wavelength of 240 nm (Figure 2).^42^ Moreover, samples without stabilizing ions and with the addition of 10 mM Na^+^ ions of KSHV, BBC3, and TRIM32 G4s showed a positive peak at 295 nm without a significant peak at 210 nm, which indicates the formation of hybrid or parallel G4s conformations.^42^ A peak shift to a wavelength of 295 nm was also observed in RelB G4 with the addition of Na^+^ ions, but not in a buffer without ions, where the spectrum indicates the predominant formation of B-like structure or random coil.^43,44^ Determining the predominant DNA conformation of ASTN2 and IκBα G4 samples was more difficult, leading us to assume that multiple secondary structure conformations are formed in different proportions. Specifically, IκBα G4 in the K^+^ ion environment showed positive peaks at 210 and 295 nm and a small negative peak at 240 nm corresponding to a potential hybrid G4 ^42^ in the probable formation of random coil or B-like structure,^43,44^ which prevailed in the samples without ions and with 10 mM Na^+^ concentration. In contrast, the ASTN2 G4 spectra at first glance appear to be characteristic of hybrid G4; however, shifts of the positive peaks to 220 and 270–280 nm and the presence of a negative peak at 240 nm indicate the possible formation of parallel G4 and B-form in the 100 mM K^+^ environment, while A-form in the ion-free environment and in the presence of 10 mM Na^+ 42^. In conclusion, the candidate G4FS from p53 and NF-κB target genes show the potential for G4 formation, adopting a topology that is strongly influenced by the different ionic environment.

The combined evaluation of ThT fluorescence, CD spectra, and native PAGE analysis, summarized in Table 2, further supports the presence of G4 structures under stabilizing conditions. The control KSHV G4 consistently exhibited high ThT values across all conditions and clear G4 signatures in both spectroscopic and electrophoretic analyses. Similarly, RelB and IκBα sequences showed a marked increase in ThT signal in the presence of K^+^ ions, accompanied by the appearance of parallel and hybrid G4 conformations as detected by CD spectroscopy. Notably, G4-associated bands were also detected by ThT staining on native gels even in the absence of stabilizing ions, although CD data indicated predominantly non-G4 or mixed conformations under these conditions. Together, these results highlight the strong ion dependence of G4 folding and demonstrate that K^+^ ions promote the formation of well-defined G4 structures

**Table 2:** Biophysical Characterization of Analyzed Sequences with G-Quadruplex-Forming Potential Derived from Human Promoter Sequences^a^

	ThT value [-]	DNA conformation	Presence of G4s on gels (staining with ThT)
**KSHV G4**	6.67 ± 0.39 (−); 13.61 ± 0.64 (Na); 13.17 ± 0.31 (K)	Parallel G4 (−); Hybrid G4 (Na); Paralel G4 + Hybrid G4 (K)	YES (−); YES (K)
**RelB G4**	4.21 ± 0.23 (−); 7.13 ± 0.44 (Na); 10.84 ± 0.30 (K)	ssDNA (−); ssDNA (Na); Parallel G4 + Hybrid G4 (K)	YES (−); YES (K)
**IkBa G4**	3.14 ± 0.10 (−); 6.10 ± 0.25 (Na); 7.17 ± 0.49 (K)	ssDNA (−); ssDNA (Na); Hybrid G4 + ssDNA (K)	YES (−); YES (K)
**TRIM32 G4**	4.07 ± 0.17 (−); 5.98 ± 0.63 (Na); 17.05 ± 0.53 (K)	Hybrid G4 + ssDNA (−); Hybrid G4 + ssDNA (Na); Parallel G4 + Hybrid G4 (K)	YES (−); YES (K)
**BBC3 G4**	1.90 ± 0.11 (−); 2.74 ± 0.42 (Na); 8.25 ± 0.45 (K)	Hybrid G4 + ssDNA (−); Hybrid G4 + ssDNA (Na); Parallel G4 (K)	YES (−); YES (K)
**ASTN2 G4**	1.78 ± 0.27 (−); 2.08 ± 0.13 (Na); 2.65 ± 0.13 (K)	Hybrid G4 + ssDNA (−); Hybrid G4 + ssDNA (Na); Hybrid G4 + ssDNA (K)	NO (−); YES (K)

a Samples were prepared without stabilizing ions (−), with sodium ions (Na), and with potassium ions (K).

### Effect of Native Constructs RE-G4FS on Yeast Basal Transcription

After the confirmation of potential G4 formation *in vitro*, we performed functional studies in the yeast isogenic system we previously developed,^45^ by constructing reporter strains carrying the native construct p53 or NF-κB RE-G4FS along with corresponding control strains; control strains were constructed by replacement of the G4 sequence with a CT_n_ sequence which does not form a G4 structure (Table 3).

**Table 3:**
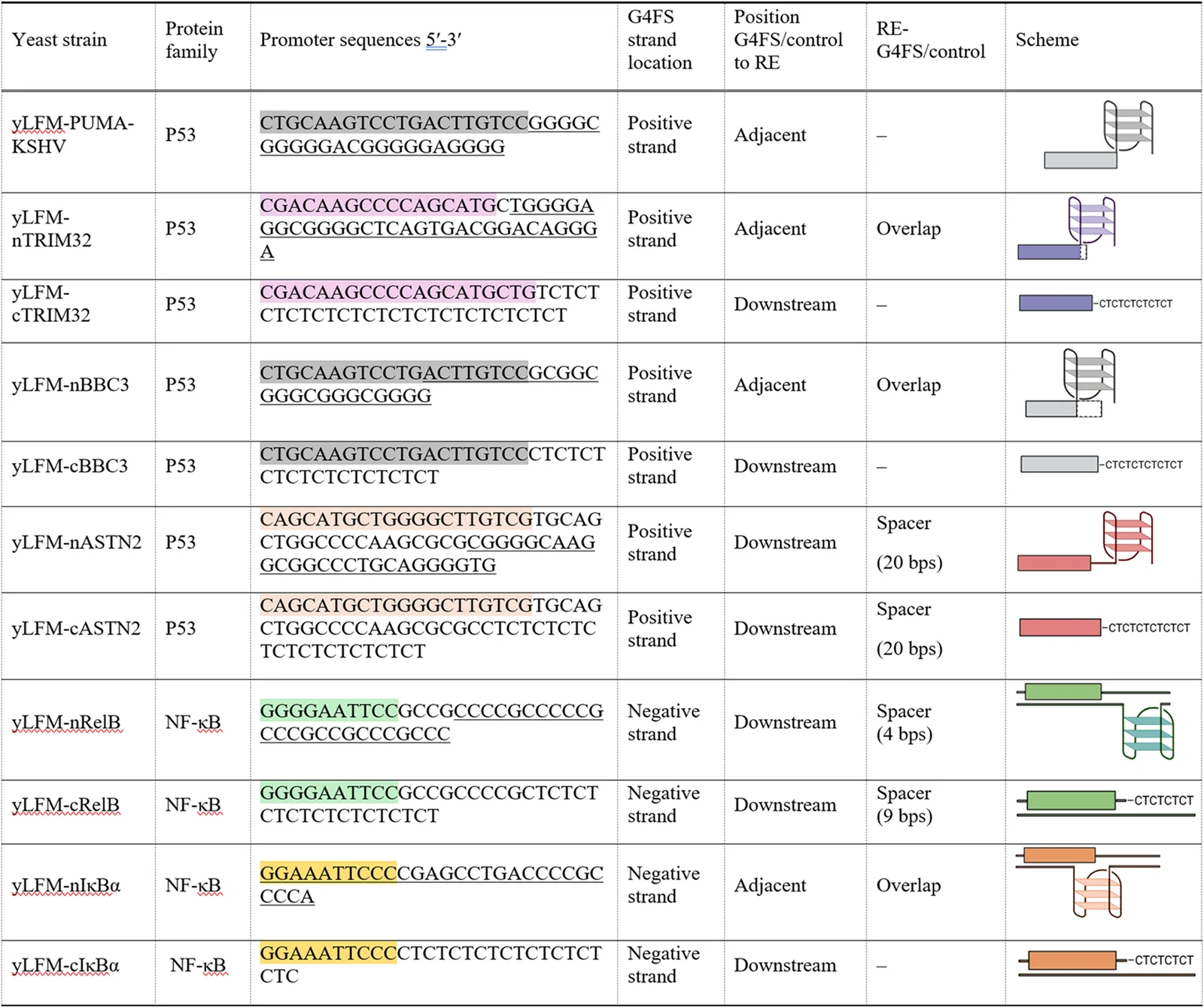
Isogenic Reporter Strains Used in the Present Study^a^

a P53 or NF-κB REs are indicated as colored boxes, while the G4FSs previously analyzed *in vitro* are underlined; CT_n_ control sequence is in bold text. The relative orientation and distance of RE with respect to G4FS or CT_n_ control sequence is also graphically represented. n = native; c = control.

First, we measured basal transcription of the endogenous yeast reporter gene by evaluating the luciferase activity of the empty vector; basal transcription was analyzed for all strains at high galactose (i.e., 1%) since we formerly demonstrated that it is slightly or not influenced by the concentration of galactose, as expected;^39^ the yLFM-PUMA-KHSV strain was used as a reference. The results showed a general enhancement of basal reporter activity in the strains containing the native RE-G4FS construct with respect to the corresponding control strains. This phenomenon was not directly correlated with G4FS features (i.e., G4 Hunter score or strand) (Figure 3).

**Figure 3:**
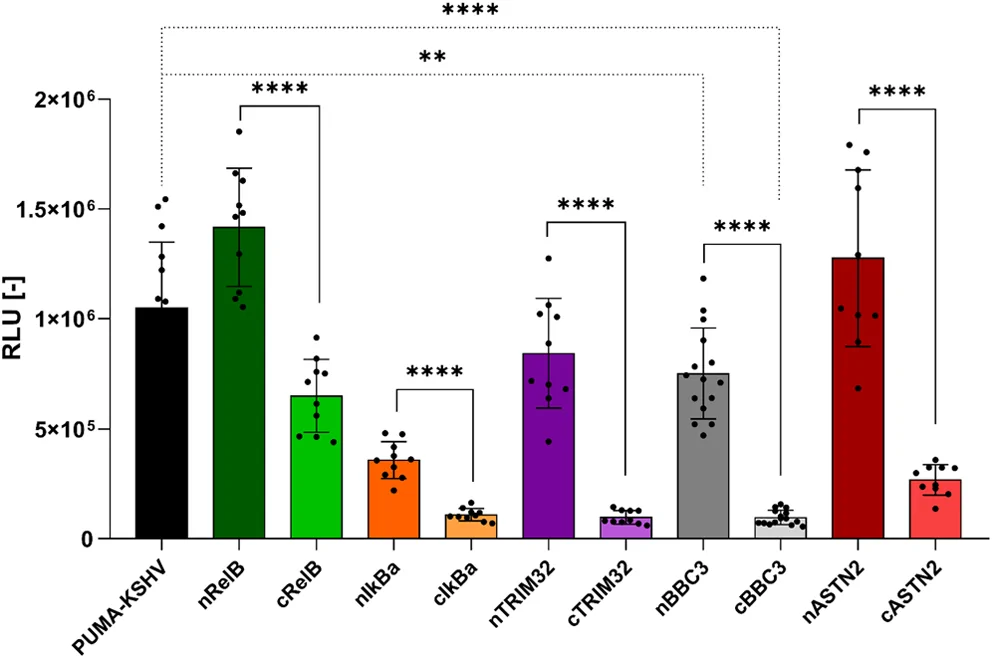
Effect of native constructs RE-G4FS from p53 and NF-κB TFs targets on basal activity in the yeast functional assay. G4FS or control sequence (CT_n_) are located downstream or adjacent from P53 or NFkB RE in isogenic yeast reporter strains. The minimal CYC1 promoter that controls *LUC1* reporter gene, provides for basal activity (see Material and Methods for details). Data were measured after 6 h of growth in medium containing 1% galactose. Results of empty vector (i.e., pRS314) in yLFM yeast strains are presented as RLUs. Bar graphs plot the average values of each measurement and standard deviations of at least 5 independent replicates; individual values are also plotted. Data were analyzed by a two-tailed Student’s *t* test. The symbols *, **, *** and **** indicate significant differences with *p* < 0.05, *p* < 0.01, *p* < 0.001 and *p* < 0.000 1; ns = not significant.

### Effect of Native RE-G4FS Constructs on p53 Family Transactivation

Then, we analyzed the effect of native RE-G4FS constructs on p53-induced transcription, by monitoring the activity of p53 family proteins in the BBC3, TRIM32, and ASTN2 strains, along with control strains. A total of six proteins were studied, including wild-type transactivation competent p53, p63, and p73 proteins (i.e., TA α variants) and p53 mutants showing partial loss of function (i.e., A161T, N235S, and R283C), for which we have previously shown varying levels of transactivation potential in the yLFM-PUMA strain.^20^ The expression levels of the studied proteins were controlled by galactose and set at high level (i.e., 1% galactose) due to the weak strength of the p53 REs under study, particularly ASTN2 and TRIM32. The results indicate that, overall, native strains expressing p53 family proteins (wild type and mutant) are associated with higher levels of reporter activity with respect to their corresponding control strains (Figure 4A), indicating that native G4FSs could modulate the activity of p53 family TFs acting at different REs, including the low-affinity ASTN2 and TRIM32 sites.

**Figure 4:**
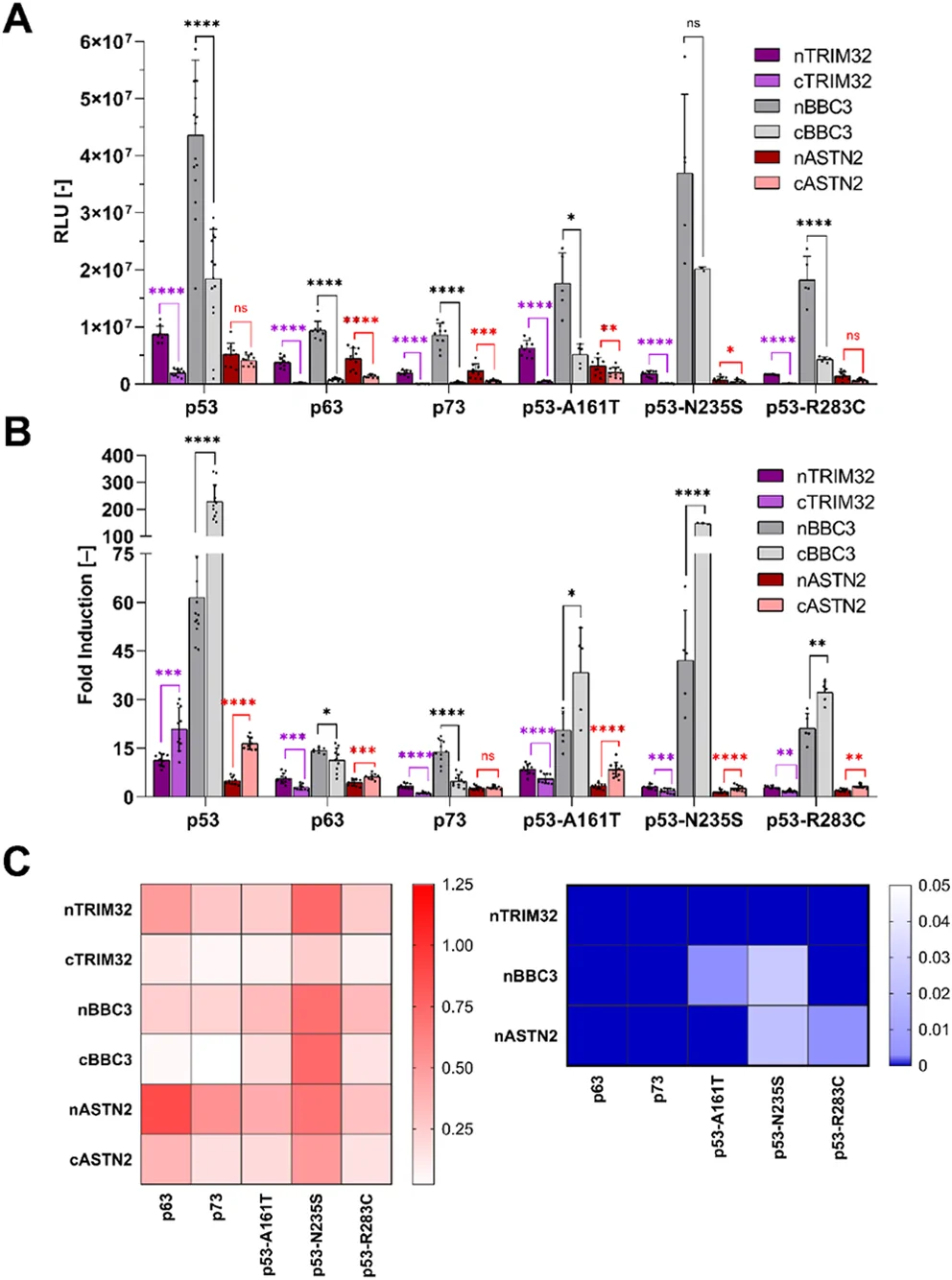
Effect of native constructs RE-G4FS on p53 family proteins transactivation in the yeast-based functional assay. Data were measured after 6 h of growth in medium containing 1% galactose. Results of p53 family proteins (i.e., p53, p63, and p73; p53 A161T, N235S, and R283C) in yLFM yeast strains (i.e., native and control strains) are presented as RLUs (A), fold induction (B), and relative activity (C). (A, B) Bar graphs plot the average value as RLU or fold induction over empty with standard deviation of at least 5 independent replicates; individual values are also plotted. Data were analyzed by two-tailed Student’s *t* test. The symbols *, **, *** and **** indicate significant differences with *p* < 0.05, *p* < 0.01, *p* < 0.001 and *p* < 0.000 1; ns = not significant. (C) Red-toned heat map plots the relative activities of p53 family proteins (i.e., p63, p73, p53 A161T, p53 N235S, and p53 R283C) compared to wild-type p53 (set to 1) and calculated as fold induction ratios. Data were analyzed by two-tailed Student’s *t* test and corresponding statistical analyses were shown in the blue-toned heat map (shades of blue, *p* value <0.05; white = not significant).

The transactivation data were also plotted as fold induction (i.e., using basal transactivation levels to normalize the impact of protein expression in each strain), highlighting a significant reduction with all reporter strains containing G4FSs for p53 wild-type protein (Figure 4B). These results are related to the effect of G4FS on the basal, p53-independent transcription of the *LUC1* reporter gene (Figure 3) and were visible and significant also for mutant p53 proteins A161T, N235S, and R283C in all strains, with the exception of the strain containing the native RE-G4FS TRIM32 construct. Regarding the other members of the p53 family, a significant reduction in fold induction was evident only for p63 in the native ASTN2 strain with respect to the control strain, possibly due to the low level of transactivation by the proteins in the remaining strains.

To focus on the relative impact of the presence of the native RE-G4FS construct on each p53 family protein, we analyzed the data as relative activity over wild-type p53. Results indicate that, overall, p53 family proteins (i.e., p63 and p73 as well as the partial function p53 mutant proteins A161T, N235S, and R283C) exhibit higher relative transactivation when G4FSs near a p53 RE are present at the promoter site with respect to the control construct (Figure 4C, red-toned heat map). The weak TFs p63 and p73 are the most positively affected by the presence of a G4FS, while, among p53 mutant proteins, the most transactivation competent N235S variant is the least affected (Figure 4C, blue-toned heat map). Our results showed that the presence of native G4s can increase the transactivation ability of those p53 family proteins characterized by weak activity.

### Effect of RE-G4FS Native Constructs on NF-κB Family Transactivation

Similarly to p53, we investigated the effect of native RE-G4FS constructs on the transactivation of NF-κB family proteins. Interestingly, in the selected RelB and IκBα target sequences, the G4FS are located on the opposite (negative) strand relative to the RE. Overall, the results did not reveal a clear higher activity in native strains over control strains when different NF-κB proteins were expressed (i.e., single expression of p65 or p50 TAD; coexpression of p65-p50 TAD or p65-p50), with the exception of p65 on native IκBα (Figure 5A). By plotting the data as fold induction, a lower value for native RE-G4FS-containing strains compared to the controls was evident (Figure 5B), consistent with the higher basal activity measured in the former (Figure 3). As previously demonstrated, p65 is a stronger transcription factor compared to p50 TAD, and their coexpression results in transactivation levels that were approximately an average of those seen with the expression of either protein; in addition, nonchimeric p50 that lacks the transactivation domain, when coexpressed with p65, leads to strong inhibition of p65-dependent transactivation. To evaluate whether GF4S may favor the transactivation of NF-κB protein combinations that are characterized by a weaker transactivation ability with respect to p65, we focused on their relative activity. The results showed that, unlike what we observed with p53 family proteins, the effect is dependent both on the type of protein expression and the RE-G4FS context (Figure 5C, red-toned heat map). Only coexpressed p65-p50 TAD proteins took advantage of the G4FS presence on the RelB construct; conversely, the absence of G4FS from the IκBα construct favored the transactivation by the NF-κB protein p50 TAD alone as well as its coexpression with p65 (Figure 5C, blue-toned heat map).

**Figure 5:**
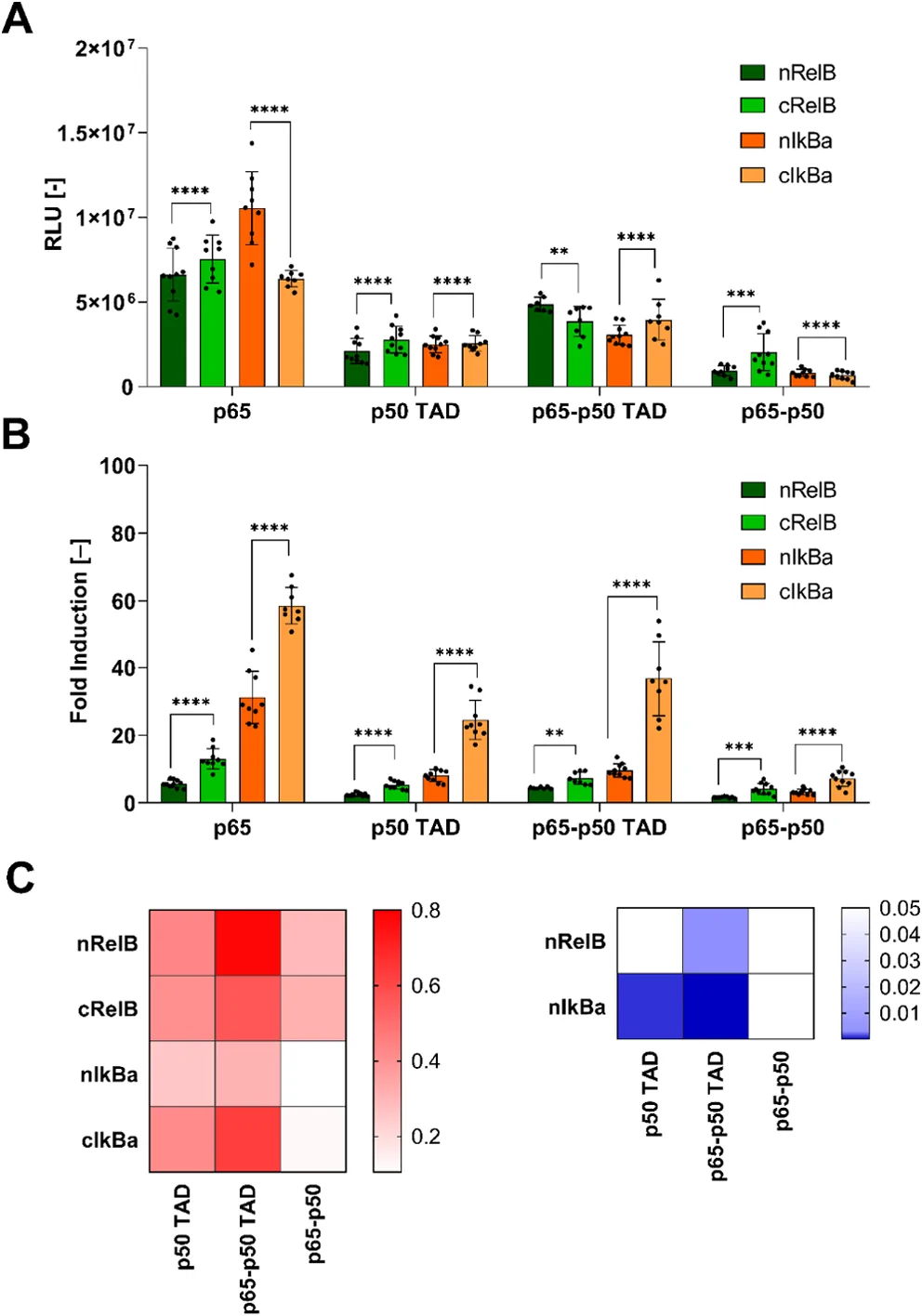
Effect of native constructs RE-G4FS on NF-κB family proteins transactivation in the yeast-based functional assay. Data were measured after 6 h of growth in medium containing 1% galactose. Results of NF-κB family proteins in yLFM yeast strains (i.e., native and control strains) are presented as RLUs (A), fold induction (B) and relative activity (C). (A, B) Bar graphs plot the average values as RLU or fold induction over empty and standard deviations of at least 5 independent replicates; individual values are also plotted. Data were analyzed by two-tailed Student’s *t* test. The symbols *, **, *** and **** indicate significant differences with *p* < 0.05, *p* < 0.01, *p* < 0.001 and *p* < 0.000 1; ns = not significant. (C) Red-toned heat map plots the relative activities of NF-κB proteins (i.e., p50 TAD, p65-p50 TAD and p65-p50) compared to p65 (set to 1) and calculated as fold induction ratios. Data were analyzed by two-tailed Student’s *t* test, and corresponding statistical analyzes were shown in the blue-toned heat map (shades of blue, *p* value <0.05; white = not significant).

## Discussion

Human TFs orchestrate gene expression through sequence-specific DNA binding, and the dysregulation of their activity is linked to human disease. Recent advances underscore the complexity of TF-DNA interactions, revealing that even low-affinity enhancer sites can contribute to gene regulation, as demonstrated by biophysical models that capture the specificity of different human TFs across a wide affinity range.^46^ Among TFs, p53 and NF-κB stand out as master regulators of cellular stress responses and, in the case of p53, also of chromatin architecture.^47^

In fact, beyond its canonical role in the initiation of transcription, p53 acts as a pioneer factor capable of binding local DNA structures and closed chromatin, and initiating epigenetic remodeling, establishing new enhancers and driving transcription at select loci.^47,48^ This dynamic interplay between DNA binding and chromatin remodeling is central to p53’s tumor-suppressive functions, which are frequently compromised in cancer. Indeed, *TP53* is the most commonly mutated gene in human malignancies, with mutations in its DNA-binding domain that alter transcriptional programs and contribute to poor clinical outcomes.^49–51^ In addition, structural alterations related to mutations in the p53 oligomerization domain can also lead to both loss-of-function and neo-morphic gain-of-function properties, reshaping cellular metabolism and apoptotic pathways.^52^ Therefore, p53 is considered an attractive therapeutic target due to its central role in the stress response and tumor suppression.^51^ The p53 regulation of BBC3, TRIM32, and ASTN2 promoters also reflects the distinct biological roles of these genes in cancer and their sensitivity to variations in p53 transcriptional competence. *BBC3* (PUMA) represents a high-threshold apoptotic target, whose efficient activation typically requires strong p53 signaling. In contrast, *TRIM32* and *ASTN2* are regulated through weaker or more context-dependent p53 response elements. Accordingly, differences among the p53 mutants analyzed, ranging from the relatively transactivation-competent N235S to the weaker DNA-contact mutant R283C, translate into distinct promoter-specific responses rather than a uniform loss of function. These observations support the notion that cancer-associated p53 mutations reshape transcriptional programs in a gene- and promoter-dependent manner. Collectively, these findings highlight the multifaceted roles of p53 in DNA recognition, chromatin remodeling, and cancer pathogenesis, while emphasizing the need to explore how the structural diversity of p53 and its binding sites influences transcriptional networks.

While our earlier studies established an isogenic yeast system to explore the transactivation of p53 as well as of the other well-known TF NF-κB in the context of isolated RE,^37,45^ here we cloned the natural human promoter region containing both the RE and the associated G4-forming sequence, including short flanking nucleotides. Then, we examined the impact of G4FS on transcriptional activity, confirming that G4FS in the promoters we selected (i.e., *BBC3*, *TRIM32* and *ASTN2* for p53 proteins family; *RelB* and *IκBα* for NF-κB family proteins) significantly influence basal transcriptional activity and modulate the transactivation activity of both families of TFs. This effect might be associated with the *in vivo* formation, in yeast cells, of G4 structures, of which we previously evaluated *in vitro* the G4 formation probability, and stability.

The transcriptional variations in p53 and NF-κB family proteins observed using the yeast assay, suggest that G4 presence alters chromatin architecture, even with G4s on the negative strand, potentially affecting nucleosome positioning and recruitment of remodeling complexes and thereby affecting promoter accessibility and transcriptional activation.^48^ We previously performed an extensive systematic mutational analyses of guanine residues, including sequential G-substitutions and rational G4-disruptive designs informed by the G4-Killer algorithm and G4Hunter scores, using model sequences derived from the well-characterized KSHV G4.^39^ These prior studies established robust correlations between guanine content, G4-forming propensity, and functional outcomes, providing a validated biophysical framework upon which the current study was built. Interestingly, in the present work we interrogate the regulatory potential of naturally occurring, promoter-embedded G4FSs in a biologically relevant transcriptional context.

Our new results confirmed that the presence of G4s increases the activity of the weakest p53 family proteins, independently from the position (i.e., adjacent or downstream); specifically, the activity of p63 and p73 was the most supported, while within p53 mutant proteins, the strongest transactivator (i.e., N235S) was less supported. These findings point to the biological relevance of G4 action mechanisms in cancer cells, where p63 and p73, may partially substitute mutant p53 and compensate for its partial or complete loss of tumor suppressor function.^53^ These findings support the concept that cancer-associated p53 mutations do not simply attenuate transcription globally but instead rewire promoter- and gene-specific regulatory programs. Our results also demonstrated the possible influence of G4FS on the activity of the NF-κB protein family, where the G4FS was located on the opposite (negative) strand relative to the transcription start site, although the effect was not as consistent as for the p53 family proteins. In fact, while in both promoters (*RelB*, *IκBα*) the presence of a G4 sequence was associated with higher basal activity, increased transactivation upon NF-κB proteins expression was observed only for selected dimer combinations (e.g., p65–p50 TAD in RelB). This phenomenon may result from a modulation of local DNA topology and/or nucleosome positioning by G4 structures, which may facilitate accessibility to the basal transcription machinery. G4FS may also enhance RE accessibility for TFs or promote the formation of protein–DNA complexes with higher stability. However, the different impact on the activity of p53 or NF-κB family proteins suggests context-dependent effects. It has been shown that G4-prone sequences are present within the binding regions of both wild-type and some p53 mutants in myeloid leukemia cell lines^19^ and that wild-type p53 may even bind to and stabilize G4 structures *in vitro* in the *c-MYC* promoter.^18^ This structure-specific recognition of G4 motifs (even at greater distances from the RE) by p53 family proteins could therefore potentially contribute to the recruitment of cooperating proteins. In contrast, NF-κB family protein activity may depend indirectly on the presence of G4s, through changes in chromatin accessibility or interactions with other proteins. Furthermore, their activity may also be influenced by changes in the topology of NF-κB protein–DNA binding.^54^ The presence of a G4 motif on the negative strand may additionally induce local torsion or changes in DNA curvature and winding that are less favorable for the p65–p50/p65–p50 TAD heterodimeric complex, potentially leading to changes in basal activity where G4s do not directly influence NF-κB-induced transcription, but instead act as local chromatin destabilizers, increasing accessibility of the region to the basal transcription machinery. Overall, the results obtained with native sequences from human promoters and the respective control strains support the idea that G4 (and possibly other noncanonical DNA structures) help to increase basal transcription of genes, independently of the position and the DNA strand containing them, suggesting that their impact is not related to the properties of the primary DNA sequence, but to the conformation and accessibility of the given site.

In conclusion, our findings suggest that G4 structures influence transcription through a combination of direct interactions with TFs and indirect effects mediated by changes in chromatin accessibility and local DNA topology. Our results expand current research on the activity of the p53 and NF-κB protein families, providing valuable insights that could be applied in the development of therapeutic approaches for cancer treatment and the study of inflammatory diseases.

Limitations of the study. Yeast reporter assays in *S. cerevisiae* provide a valuable and highly controlled system to assess the functional consequences of sequences capable of forming G4 structures; however, some limitations should be acknowledged. First, the yeast assay measures changes in reporter gene expression rather than directly detecting G4 formation *in vivo*, and thus the observed transcriptional effects should be interpreted as indirect indicators of G4 activity. Second, although the reporter constructs are genomically integrated, they are analyzed outside of their full native chromosomal context, and therefore may not fully recapitulate all higher-order chromatin features, long-range regulatory interactions, or human-specific cofactors that influence G4 formation and transcriptional regulation in mammalian cells. In the present study, we attempted to mitigate this limitation by evaluating G4-forming sequences together with short native flanking regions derived from human promoters, rather than using minimized synthetic constructs as in our previous work,^20,39^ thereby increasing the biological relevance of the present results.

## Supplementary Material





## Notes

Supporting Information 01: G4Hunter analyzes and ChIP-Atlas Enrichment Analysis (XLSX)
Supporting Information 02: Biophysical characterization of G4 oligonucleotides including samples with lithium ions and Supporting Information 03: G4 structures detected on nondenaturing polyacrylamide gel electrophoresis (PDF)
