# Supplementary material for: An Isogenic Yeast Model Reveals the Influence of Native Promoter G‑Quadruplex Forming Sequences on P53 and NF-κB Transactivation
Source: ACS Bio Med Chem Au. 2026 Jul 4;6(4):456–67. doi: 10.1021/acsbiomedchemau.6c00109 (PMC13495015; doi:10.1021/acsbiomedchemau.6c00109)
Supplement: Supplementary file 1 [file bg6c00109_si_002.pdf]

An isogenic yeast model reveals the influence of native promoter G-quadruplex forming sequences on P53 and NF-κB transactivation

Libuše Kratochvilová[1,2], Petr Orság[1], Karolína Drápalová[1,2], Radim Stříž[2], Zeinab El Rashed[3], Paola Menichini[4], Rieko Ohki[5], Paola Monti[4], Alberto Inga[6*], Václav Brázda[1,2*§]*

[1] Institute of Biophysics of the Czech Academy of Sciences, Královopolská 135, 61200 Brno, Czech Republic

[2] Department of Food Chemistry and Biotechnology, Faculty of Chemistry, Brno University of Technology, Purkyňova 118, 61200 Brno, Czech Republic

[3] Gene Expression Regulation, IRCCS Azienda Ospedaliera Metropolitana, 16132 Genoa, Italy

[4] Neuro-oncology and Mutagenesis, IRCCS Azienda Ospedaliera Metropolitana, 16132 Genoa, Italy

[5] Laboratory of Fundamental Oncology, National Cancer Center Research Institute, 5-1-1 Tsukiji, Chuo-ku Tokyo, 104-0045, Japan

[6] Laboratory of Transcriptional Networks, Department of Cellular, Computational and Integrative Biology, CIBIO, University of Trento, via Sommarive 9, 38123 Trento, Italy

§Email: vaclav@ibp.cz

Supplementary materials

Supplementary material 02: Biophysical characterization of G4 oligonucleotides including samples with lithium ions.

Supplementary material 03: G4 structures detected on nondenaturing polyacrylamide gel electrophoresis.

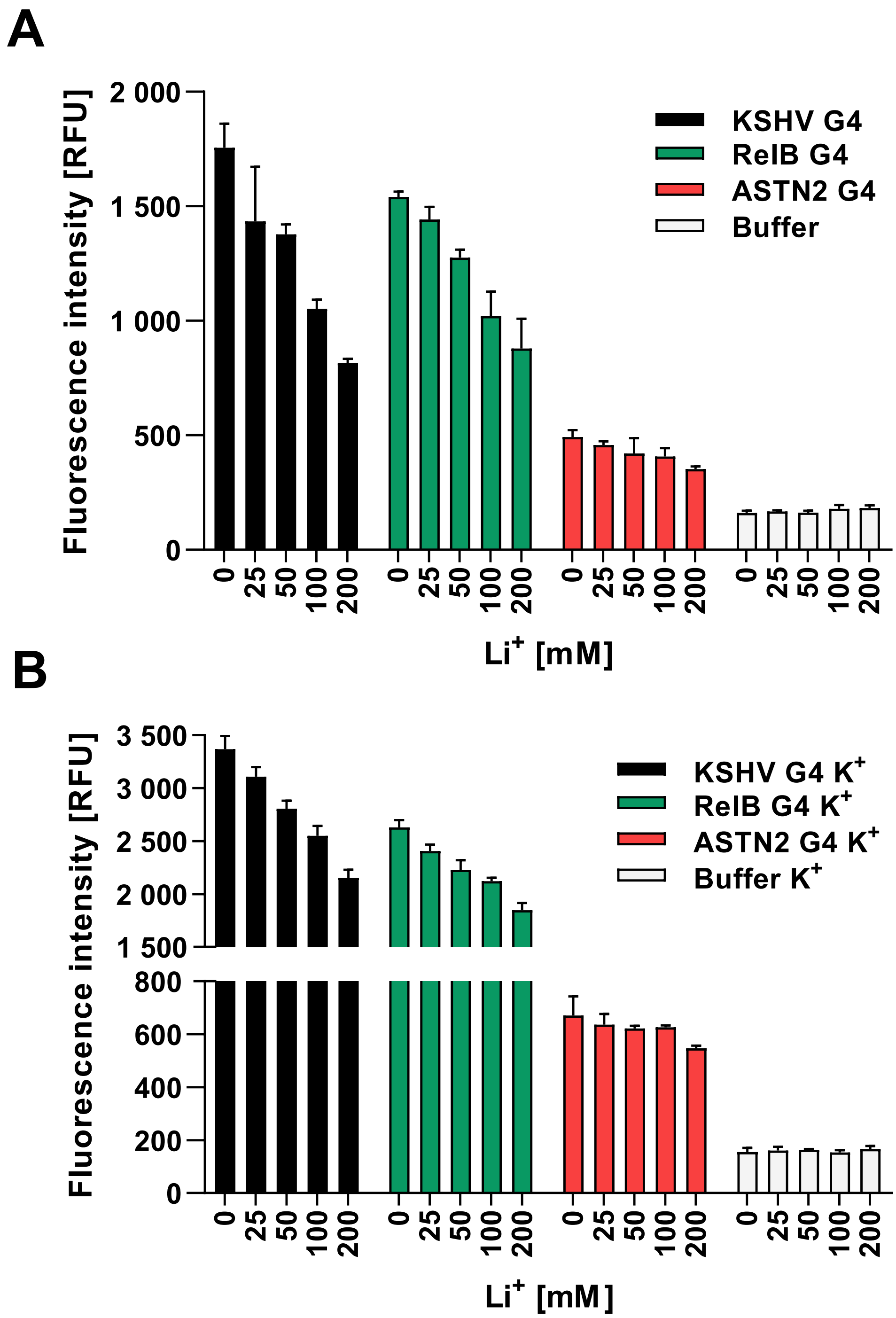

Supplementary material 02. *ThT with selected G4FSs supplemented with Li+ ions. DNA samples of selected G4FSs with the highest (RelB G4) and lowest (ASTN2 G4) G4Hunter scores, together with the positive control KSHV G4, at a concentration of 4 µM, were hybridized either without addition* ***(A)*** *or with the addition of 200 mM KCl* ***(B)****, under the same conditions as in the ThT assay, overnight. The following day, the DNA samples were mixed 1:1 with buffer containing Li^{+} ions (0–50–100–200–400 mM; the final concentrations of all components were therefore halved and corresponded to the concentrations used in the ThT assay in Figure 2). The samples were subsequently analyzed in the same manner as in the ThT assay in Figure 2.*

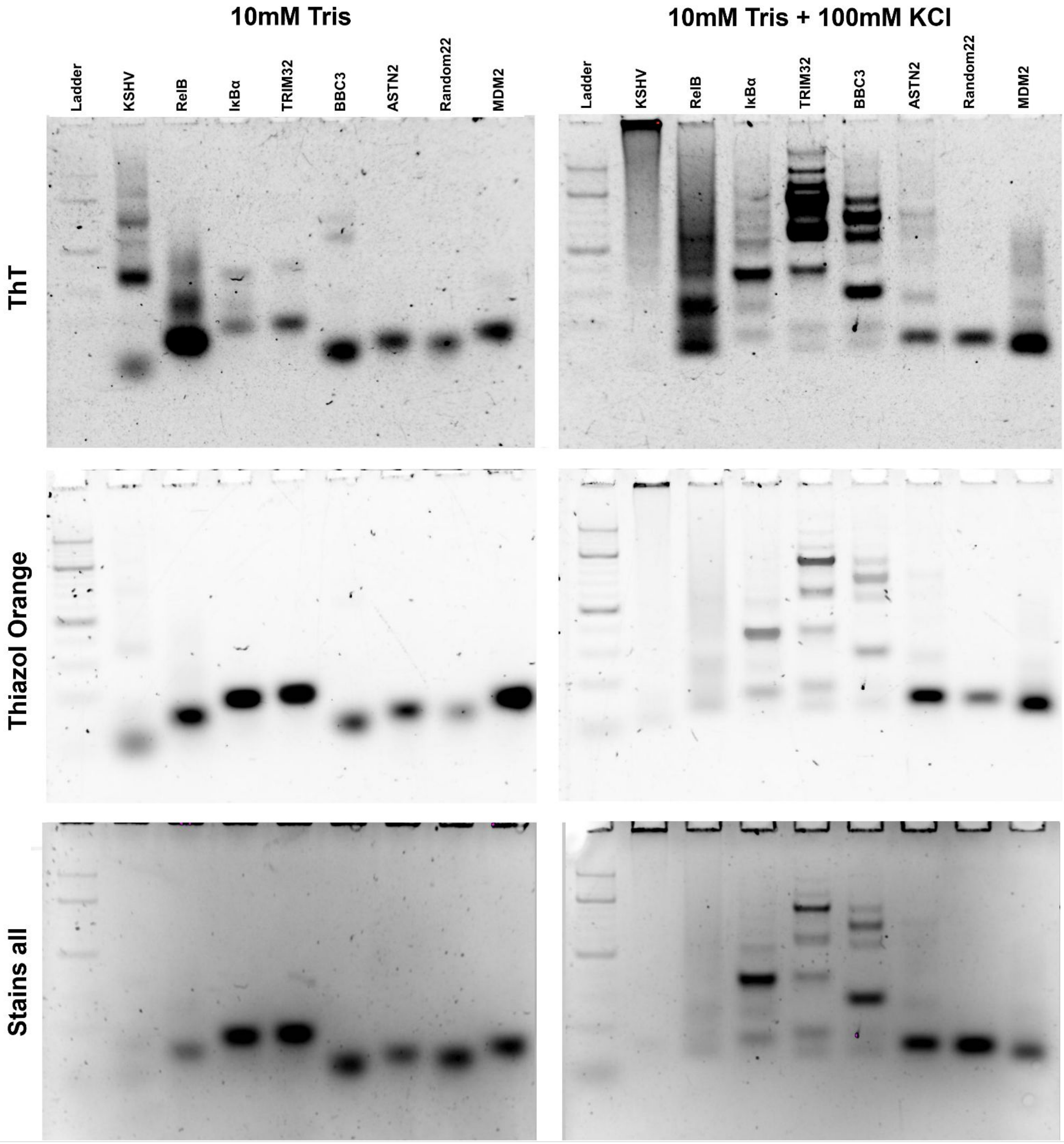

Supplementary material 03. *G4 structures detected on nondenaturing polyacrylamide gel electrophoresis by ThT, Thiazol Orange and Stains all staining. The six different oligonucleotides with potential to form G-quadruplex structure, negative control sequence (non-G4FS ssDNA; 5´-CCGAGCGATTAAGCGTGACAGC-3´; 22 bases) and control G4FS MDM2 (positive control forming in vitro G4s)* [1] *were tested at 10 µM concentration. Samples were prepared in a 10 mM Tris with or without 100 mM KCl and loaded on a nondenaturing 8% acrylamide gel. The gels were electrophoresed at 4 °C with the migration marker O'RangeRuler™ 10 bp DNA Ladder. The same gels were visualized by gradually staining with 0.5 µM ThT, 2.73 µM Thiazol Orange and with Stains all. Staining of G4 structures by ThT (upper panel), Thiazol Orange (middle panel) and Stains all (lower panel).*

(1) *Kratochvilová, L.; Dinová, A.; Valková, N.; Dobrovolná, M.; Sánchez-Murcia, P. A.; Brázda, V. Chromatin Immunoprecipitation Reveals P53 Binding to G-Quadruplex DNA Sequences in Myeloid Leukemia Cell Lines. ACS Bio Med Chem Au* ***2025****, 5 (2), 283–298. https://doi.org/10.1021/acsbiomedchemau.4c00124.*
